# Supplementary figures and images for: Application of a Novel Quantitative Trait Locus Combination to Improve Grain Shape without Yield Loss in Rice (Oryza sativa L. spp. japonica)
Source: Plants (Basel). 2023 Mar 30;12(7):1513. doi: 10.3390/plants12071513 (PMC10097285; doi:10.3390/plants12071513)

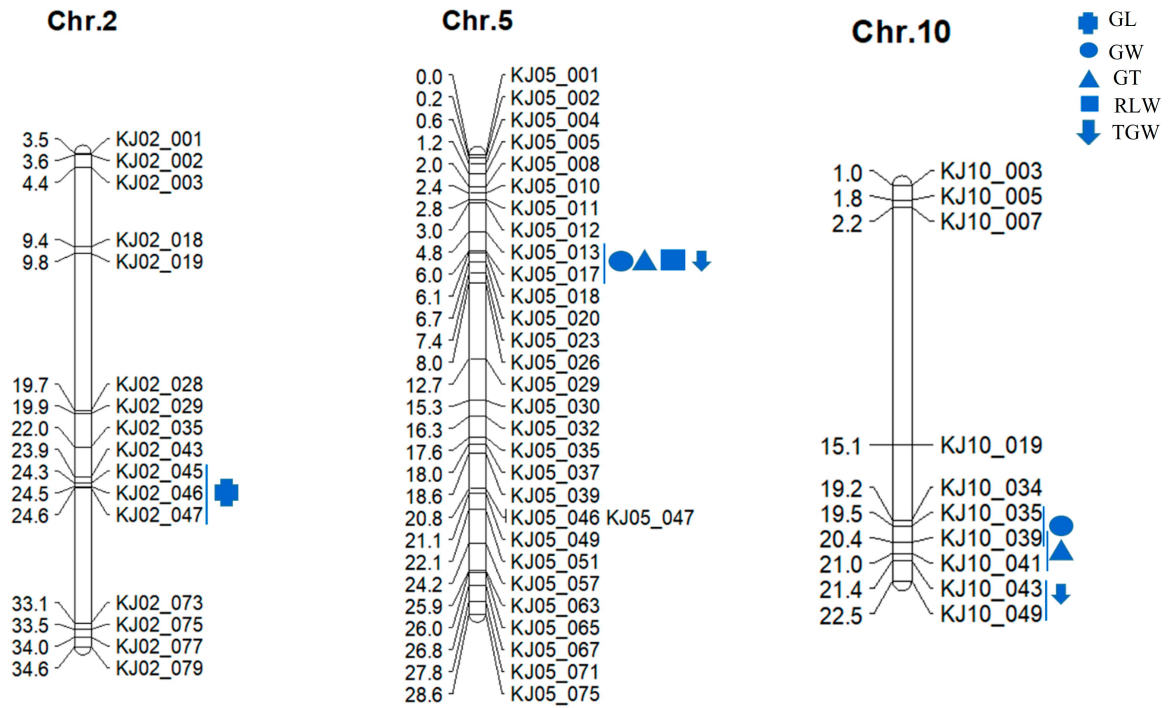

**Figure S1.** Positions of detected QTLs associated with grain shape tested on chromosomes 2, 5, and 10.

Supplement: Supplementary file 1 [file plants-12-01513-s001.zip › plants-2285920-Figure S1.pdf]
